# Integrating whole-exome and genotyping array data for pharmacogenetic typing: a comparative evaluation with whole-genome sequencing in UK Biobank

**DOI:** 10.3389/fphar.2026.1892708

**Published:** 2026-10-26

**Authors:** Chanhee Lee, Seokho Jeong, Joo-Yeon Lee, Jimin Lee, Jong Hyun Park, Joohon Sung, Seunggeun Lee, Minjung Kho

**Affiliations:** 1 Graduate School of Data Science, Seoul National University, Seoul, Republic of Korea; 2 Institute of Health and Environment, Seoul National University, Seoul, Republic of Korea; 3 Graduate School of Public Health, Seoul National University, Seoul, Republic of Korea

**Keywords:** bioinformatics, genomics, haplotype, pharmacogenetics, pharmacogenomics, precision medicine

## Abstract

**Introduction:**

Whole‐genome sequencing (WGS) provides comprehensive coverage of coding, non‐coding, and rare variants for pharmacogenetic star‐allele typing, but cost and computational demands limit its population-scale use. Whole-exome sequencing (WES) and imputed genotyping arrays are more widely available, yet their WGS-referenced performance remains incompletely defined.

**Methods:**

We benchmarked star-allele calls from WES, imputed array data, and an integrated call set combining WES and array data against an Aldy-derived WGS analytical reference across 33 pharmacogenes in 1,450 multi-ancestry UK Biobank participants. We also developed PGxIntegrator, an open‐source workflow that harmonizes WES and array data and enables scalable Aldy‐based star‐allele calling.

**Results:**

Using WES or imputed array data alone, 25 of 33 genes reached high diplotype concordance with the WGS analytical reference (≥95%); the integrated call set also achieved ≥95% concordance for 25 genes and improved or maintained concordance relative to the better‐performing single platform for 29 genes overall. The largest incremental gains among high‐concordance loci were observed for CYP1A1, CYP3A4, and DPYD, where integration improved concordance by 1.3–3.3 percentage points over the better‐performing single platform, consistent with complementary recovery of non-coding and rare haplotype‐defining variants. In contrast, UGT1A1 and CYP2D6 remained challenging despite integration.

**Discussion:**

For CYP2D6, the WGS analytical reference was generated by a single computational caller and was not orthogonally validated; concordance therefore reflects agreement with this reference rather than clinical typing accuracy. These findings provide practical gene‐level guidance for population‐scale pharmacogenetic analysis when WGS is unavailable.

## Introduction

Pharmacogenetics plays a critical role in precision medicine by tailoring drug therapy based on individual genetic variation (Mosch et al., 2025; Halman et al., 2024). In clinical settings, pharmacogenetic testing has traditionally relied on single-gene or small multi-gene panels targeting variants with well-established clinical utility. These targeted approaches remain widely used to guide medication selection, dosing, and monitoring—particularly for drugs such as warfarin, clopidogrel, and thiopurines—owing to their lower cost and rapid turnaround time (Mosch et al., 2025). However, these targeted panels are inherently limited in scope and often fail to capture rare or structural variants that may influence drug response.

With the increasing accessibility of sequencing technologies, there is growing interest in genome-wide pharmacogenetic analysis that can capture the full spectrum of relevant variation (Yang et al., 2016; Caspar et al., 2021; Cohn et al., 2017). Whole-genome sequencing (WGS) is particularly promising in this regard, offering comprehensive coverage across both coding and non-coding regions, as well as the ability to detect rare and structural variants (Yang et al., 2016; Caspar et al., 2021; Cohn et al., 2017). Yet, despite falling costs, the widespread implementation of WGS in research and clinical settings remains constrained by its data intensity, processing demands, and overall cost.

In contrast, whole-exome sequencing (WES) and genotyping arrays are already widely available across large biobank cohorts (Szustakowski et al., 2021). While WES offers high-resolution coverage of coding regions, it often misses non-coding regulatory variants relevant to pharmacogenes. Genotyping arrays provide genome-wide variant information at relatively low cost, but rely on imputation and do not directly capture read-level features such as sequencing depth or structural variation signals (Yang et al., 2016; Caspar et al., 2021). Similarly, although raw read WES provides high-confidence variant calls in coding regions, VCF-based inputs from either WES or array data remain limited for pharmacogenetic star-allele calling at complex loci because they do not retain the full information required to resolve copy number variation, structural rearrangements, and phasing (Halman et al., 2024; Hari et al., 2023).

To address these gaps, McInnes et al. applied PGxPOP to United Kingdom Biobank (UKBB) data and constructed an integrated call set that combined coding variation from exome sequencing with non-coding variation from imputed genotyping arrays (McInnes et al., 2021). Their work demonstrated the feasibility of large-scale PGx profiling and revealed substantial discordance among exome, imputed array, and integrated call sets for 14 pharmacogenes, consistent with complementary limitations of the individual platforms. However, because the analysis was not benchmarked against an external high-resolution reference such as WGS, it did not establish gene-specific concordance with a high-resolution analytical reference or systematically identify which pharmacogenes benefit most from integration.

In this study, we extend prior efforts by systematically benchmarking star-allele calls from WES, array, and their integration against Aldy-derived WGS calls as an analytical reference, across 33 pharmacogenes in 1,450 UKBB participants of diverse ancestries. To support this effort, we developed PGxIntegrator, a harmonized and reproducible pipeline for integrated star-allele typing from WES and array data. Our findings quantify the strengths and limitations of integration, identify gene-level performance differences, and highlight the potential of this approach for scalable pharmacogenetic analysis when WGS is not yet feasible.

## Materials and methods

### Study cohort and data sources

We used genomic data from the United Kingdom Biobank (UKBB), a large-scale prospective cohort of approximately 500,000 individuals aged 40–69 at recruitment, designed to facilitate population-scale biomedical research (Sudlow et al., 2015). For this study, we analyzed a subset of 1,450 participants with all three genomic data types available: WGS, WES, and imputed genotyping array data. Stratified random sampling was applied across ancestry groups to obtain a more balanced representation within the study cohort. The final cohort consisted of individuals spanning four major ancestry groups based on self-reported ethnicity (UKBB Data-Field 21,000): 1,000 European (EUR) participants (code 1001: British), 150 East Asian (EAS) (code 5: Chinese), 150 African ancestry (AFR) participants (code 4002: African), and 150 South Asian ancestry (SAS) participants (codes 3001: Indian, 3002: Pakistani, or 3003: Bangladeshi). UKBB received ethical approval from the North West Multi-centre Research Ethics Committee, and all participants provided informed consent. This study used de-identified United Kingdom Biobank data accessed under application number 45227. The present analysis was conducted using secondary data and did not involve direct participant contact.

The majority of samples in this study were of EUR ancestry, reflecting the underlying composition of the UKBB cohort. As such, overall concordance metrics are largely influenced by performance in EUR individuals. To account for this, we stratified all analyses by ancestry group (Supplementary Table S1).

WGS data were generated by UKBB using the Illumina NovaSeq 6000 platform and processed using the UKBB Research Analysis Platform (RAP) pipeline following GATK best practices (Li et al., 2023). Reads were aligned to the GRCh38; preprocessing included duplicate marking, base quality recalibration, and variant calling. Samples were retained only if they demonstrated <2% mismatch rate with genotyping array data, ensuring sample-level concordance. WES was performed on 454,787 participants using the IDT xGen Exome Research Panel v1.0 and also sequenced on the NovaSeq 6000 platform (Backman et al., 2021). Variant calling was conducted through the Original Quality Functionally Equivalent (OQFE) pipeline, which uses weCall (Backman et al., 2021) to generate gVCFs and GLnexus to create joint multi-sample VCFs (Backman et al., 2021). After genotype-level QC (excluding genotypes with depth <10), mean read depth across SNP sites within each pharmacogene in the WES VCF ranged from 17.2 to 40.9 (Supplementary Table S2), providing gene-level coverage context for WES-based star-allele calling (Backman et al., 2021). Genotyping array data were derived from the UK BiLEVE Axiom Array (∼50K samples) and the UKBB Axiom Array (∼450K samples), encompassing ∼820,000 markers. Genotypes were imputed using IMPUTE4 with the Haplotype Reference Consortium (HRC) reference panel (39235157 markers), aligned to GRCh37 (Bycroft et al., 2018). Additional technical details can be found in the original UKBB documentation and cited studies.

### PGxIntegrator: an integrated pipeline for data harmonization and pharmacogenetic variant analysis

As illustrated in Figure 1, each data type underwent preprocessing to ensure consistency across platforms prior to integration and pharmacogenetic analysis.

**FIGURE 1:**
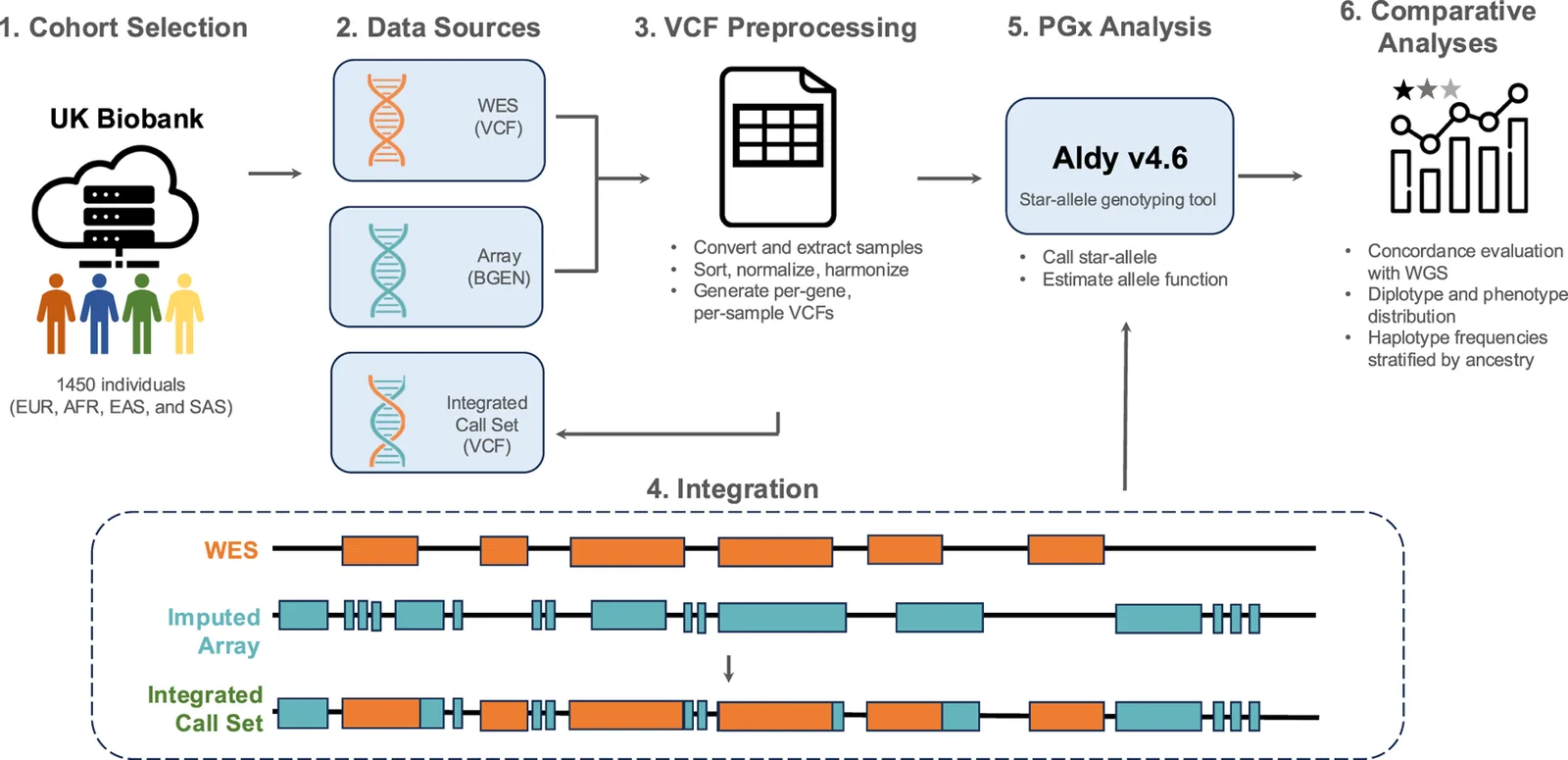
Overview of the Integrated Pharmacogenomic Typing Pipeline A total of 1,450 individuals from the UK Biobank (African, East Asian, European, South Asian) were selected. WES (VCF) and imputed array (BGEN) data were processed and harmonized to generate an integrated VCF. Per-gene, per-sample VCFs were created for star-allele calling using Aldy. Post-analyses included concordance with WGS, and ancestry-stratified haplotype frequencies. The integrated call set was constructed at the normalized variant-record level by retaining the WES record when the same record was present in both datasets and incorporating array-derived records only when the corresponding record was absent from WES. The lower panel illustrates the complementarity and combined coverage across platforms.

Imputed genotyping array data were provided in BGEN v1.2 format and converted to VCF using PLINK2 (Chang et al., 2015). Standardization steps included normalizing chromosome nomenclature, standardizing variant identifiers, and reordering sample columns. Since the array data were aligned to the GRCh37, we converted coordinates to GRCh38 using UCSC LiftOver with appropriate chain files (Hinrichs et al., 2006). During LiftOver, each source variant was tracked using a CHROM:POS:REF:ALT key retained in the BED identifier field. Lifted coordinates were matched back to the original VCF records using this key rather than rsID or record order. LiftOver quality control was performed by comparing the input BED records with the mapped and unmapped outputs for each pharmacogene-specific array VCF. Variants without a primary-assembly GRCh38 mapping were excluded before downstream VCF sorting, indexing, and integration.

WES data were obtained as multi-sample VCFs. As an additional post sample quality control, we applied genotype-level filters for allelic depth (<10) and genotyping quality (<10). We then applied variant-level filters to remove sites with high missingness (>5%) and deviation from Hardy–Weinberg equilibrium (P < 1 × 10^−15^). All VCFs were left-aligned, normalized, and decomposed into biallelic records using bcftools (Danecek et al., 2021). After decomposition, entire records whose sole ALT allele was the spanning-deletion symbol *, indicating a position overlapped by an upstream deletion, were removed. This filtering removed spanning-deletion placeholder records rather than the corresponding upstream deletion variants represented using standard REF and ALT alleles. To contextualize gene-level differences in WES-based star-allele performance, we summarized mean sequencing depth across pharmacogenes using SNP sites observed in the WES VCFs (Supplementary Table S2). Gene-specific, sample-level variant files were generated for downstream harmonization and star-allele calling using custom Bash and Python scripts.

To generate an integrated call set, we merged quality-controlled variants from WES and array data. After genotype- and variant-level quality control, integration was performed at the normalized variant-record level. When the same normalized variant record was present in both WES and array data, the WES record was retained for all samples. Because WES sites with >5% missingness had already been excluded, any residual missing WES genotypes occurred at retained sites with ≤5% missingness and were left as missing rather than backfilled from the array on a sample-by-sample basis. Array-derived records were incorporated only when the corresponding normalized variant record was absent from the WES call set. Thus, PGxIntegrator does not statistically impute missing genotypes or substitute individual WES genotypes with array calls; rather, it integrates quality-controlled observed variant records from complementary platforms. Supplementary Table S3 summarizes platform-specific coverage across pharmacogenes (the number of unique variant positions identified by each platform). The resulting merged VCFs were organized by gene and sample and used for downstream star-allele inference. Apart from the integrated call sets produced by the pipeline, WGS data in CRAM format were directly utilized for high-resolution star-allele calling to serve as the analytical reference for concordance benchmarking of the harmonized results. The analytical workflow combined upstream quality-control scripts with PGxIntegrator. Study-specific genotype- and variant-level filters, including depth, genotype quality, missingness, and Hardy–Weinberg equilibrium criteria, were applied before integration. PGxIntegrator was then used for data harmonization, normalization, WES–array integration, generation of gene- and sample-specific VCFs, and parallelized Aldy-based star-allele calling. The PGxIntegrator workflow is available at [https://github.com/achl1990/PGxIntegrator]. Generative artificial intelligence tools were used in a limited manner to assist with code polishing and refactoring of author-developed scripts used in the PGxIntegrator workflow. The analytical design, variant-processing logic, quality-control criteria, and interpretation of results were determined by the authors, and all code changes and outputs were reviewed and validated by the authors.

### High-throughput star-allele typing

We performed pharmacogenetic haplotype calling using Aldy v4.6, a rule-based genotyper supporting star-allele inference across a broad range of pharmacogenes (Hari et al., 2023; Twesigomwe et al., 2020). WES, imputed-array, and integrated calls were generated from harmonized VCF inputs, whereas WGS calls were generated directly from CRAM files containing read alignments. From CRAM input, Aldy can use read-level coverage, alignment, and locus-specific evidence in its copy-number and structural-configuration models; these Aldy-derived WGS calls were used as the analytical reference. For *CYP2D6*, all equally likely WGS solutions were retained, and repeated-copy, tandem-star, hybrid- or conversion-associated, *5 deletion, and suballelic notation were preserved. No independent *CYP2D6* CNV caller or orthogonal structural assay was applied; total copy number, allele-specific copy number, hybrid breakpoints, and gene-conversion breakpoints were not independently estimated outside Aldy. In contrast, WES, imputed-array, and integrated calls used Aldy’s predefined two-copy VCF configuration and were not treated as CNV-aware *CYP2D6* analyses. The hg38 version of the Aldy v4.6 database was used for compatibility with GRCh38 coordinates. Gene-specific annotation sources are summarized in Supplementary Table S4.

To enable large-scale pharmacogenetic analysis across 33 genes in 1,450 individuals, we implemented parallel execution of Aldy using GNU Parallel (Tange, 2011). Multi-sample VCFs were split into per-sample, per-gene files, and star-allele calling was run concurrently across available CPU cores, enabling significantly reduced computational time compared to Aldy’s single-threaded operation, thereby improving scalability for population-level analyses.

### Evaluation of platform concordance

We assessed concordance of star-allele typing across WES, imputed array, and integrated datasets using the Aldy-derived WGS analytical reference. For each individual and gene, diplotype concordance was defined by exact matching of normalized Aldy Major-field solutions, with the order of the two “/”-separated haplotypes treated as phase-invariant while the internal order of “+”-joined components was preserved. When multiple equally likely solutions were reported, all complete solutions were retained, and a sample–gene pair was considered concordant if at least one normalized test solution exactly matched a normalized WGS solution; no component-level partial matching was performed. Detailed normalization rules and pseudocode are provided in the Supplementary Material.

As a sensitivity analysis, any WES, imputed array, or integrated call with more than one equally likely diplotype solution was classified as discordant, regardless of whether one candidate solution matched the WGS analytical reference. Concordance was then recalculated using the same gene-specific WGS-callable denominators as in the primary analysis (Supplementary Table S5).

Concordance was computed as the proportion of matching diplotypes among samples with a callable WGS analytical-reference diplotype for that gene. Because WGS-based Aldy calls were unavailable for a subset of samples at certain loci, concordance denominators were defined on a per-gene callable basis. Specifically, 4 samples lacked callable WGS *CYP2D6* diplotypes because Aldy failed to phase despite evidence of copy-number variation with high read depth, and 710 samples lacked callable WGS *GSTM1* diplotypes because of low coverage. These samples were retained in the cohort but excluded from gene-specific concordance denominators. Ancestry-stratified concordance was measured for each ancestry group (EUR, AFR, EAS, SAS).

### Population-stratified haplotyping

To assess the distribution of pharmacogenetic variation across ancestries, we stratified star-allele calls by ancestry group. For each gene and platform, we computed allele frequencies within each ancestry group.

### Union-of-FAIL sample definition and sankey visualization

To visualize platform-specific discordance patterns, we constructed gene-specific “union-of-FAIL” subsets consisting of individuals with a callable WGS analytical reference whose diplotype call from at least one non-WGS data source (WES, imputed array, or the integrated call set) did not match any retained WGS solution. Diplotype transitions across data sources were summarized using Sankey diagrams, with link widths proportional to the number of individuals. For readability, diplotype categories were rank-collapsed within each gene-specific union-of-FAIL subset: we retained the 18 most frequent diplotypes across all stages pooled (WES, array, integrated, WGS) and collapsed all remaining diplotypes into an “Other” category; “NoCall” was retained as a separate category regardless of frequency.

## Results

### Overall concordance patterns

We evaluated star-allele diplotype concordance across 33 pharmacogenes using the Aldy-derived WGS analytical reference, with concordance calculated on a per-gene callable basis. For the integrated call set, concordance across genes ranged from 18.2% to 100.0%, with a median of 99.0% (mean, 89.1%; interquartile range, 3.1%). Using WES or imputed array data alone, 25 of the 33 genes reached ≥95% concordance with WGS on at least one single platform. The integrated call set achieved ≥95% concordance for the same 25 genes, whereas five genes (*CYP2B6*: 47.4%*, CYP2D6*: 51.8%*, CYP2F1*: 40.1%*, GSTM1*: 18.2%*, UGT1A1*: 44.2%) showed concordance below 60%. Table 1 summarizes concordance across WES, imputed array, and the integrated call set, and Table 2 identifies the best-performing platform for each gene.

**TABLE 1:** Concordance (%) of WES, imputed array, and integrated call set with WGS analytical-reference star-allele diplotype calls across 33 pharmacogenes in the 1,450-participant UK Biobank cohort.

Gene	WES	Array	Integrated call set
*CYP1* family			
*CYP1A1*	71.9	97.9	**99.5**
*CYP1A2*	**100.0**	99.7	**100.0**
*CYP2* family			
*CYP2A13*	**99.7**	98.6	**99.7**
*CYP2A6*	**85.9**	82.1	**85.9**
*CYP2B6*	46.1	37.4	**47.4**
*CYP2C8*	**99.7**	98.6	**99.7**
*CYP2C9*	**99.4**	97.3	**99.4**
*CYP2C19*	64.9	**97.8**	**97.8**
*CYP2D6*	51.9	**54.6**	51.8
*CYP2E1*	50.1	96.4	**96.6**
*CYP2F1*	34.9	**40.1**	**40.1**
*CYP2J2*	85.4	99.0	**99.2**
*CYP2R1*	**100.0**	99.9	**100.0**
*CYP2S1*	**99.9**	**99.9**	**99.9**
*CYP2W1*	**99.9**	99.2	**99.9**
*CYP3* family			
*CYP3A4*	92.3	98.6	**99.9**
*CYP3A5*	**96.8**	96.1	**96.8**
*CYP3A7*	**100.0**	99.4	**100.0**
*CYP3A43*	99.6	**99.7**	99.6
*CYP4* family			
*CYP4F2*	97.6	99.0	**99.2**
Glutathione S-Transferases			
*GSTM1*	**18.2**	**18.2**	**18.2**
*GSTP1*	98.1	**99.6**	98.1
UDP-glucuronosyltransferases			
*UGT1A1*	26.6	**44.2**	**44.2**
*UGT2B7*	22.0	98.6	**98.8**
N-Acetyltransferases			
*NAT1*	49.7	93.0	**93.4**
*NAT2*	82.2	83.0	**84.1**
Others			
*CFTR*	**97.9**	97.3	**97.9**
*DPYD*	95.7	94.8	**99.0**
*IFNL3*	47.9	**99.9**	**99.9**
*NUDT15*	**98.3**	97.2	**98.3**
*SLCO1B1*	30.0	**98.1**	97.2
*TPMT*	**99.4**	98.8	**99.4**
*VKORC1*	12.6	98.8	**99.0**

Concordance was calculated using gene-specific callable WGS analytical-reference denominators; *CYP2D6* included 1,446 participants and *GSTM1* included 740, while the remaining genes included 1,450.

Bold values indicate the highest diplotype concordance among the three data sources for each gene. Tied highest values are also shown in bold.

**TABLE 2:** Gene-level summary of the best-performing data source (WES, imputed array, or integrated call set) for WGS-concordant star-allele typing across 33 pharmacogenes.

Data type	Genes
WES	*CFTR, CYP1A2, CYP2A13, CYP2A6, CYP2C8, CYP2C9, CYP2R1, CYP2S1, CYP2W1, CYP3A5, CYP3A7, GSTM1, NUDT15, TPMT*
Imputed array	*CYP2C19, CYP2D6, CYP2F1, CYP2S1, CYP3A43, GSTM1, GSTP1, IFNL3, SLCO1B1, UGT1A1*
Integrated call set	*CFTR,* ***CYP1A1*** *, CYP1A2, CYP2A13, CYP2A6,* ***CYP2B6*** *, CYP2C19, CYP2C8, CYP2C9,* ***CYP2E1*** *, CYP2F1,* ***CYP2J2*** *, CYP2R1, CYP2S1, CYP2W1,* ***CYP3A4*** *, CYP3A5, CYP3A7,* ***CYP4F2*** *,* ***DPYD*** *, GSTM1, IFNL3,* ***NAT1*** *,* ***NAT2*** *, NUDT15, TPMT, UGT1A1,* ***UGT2B7*** *,* ***VKORC1***

WES, whole-exome sequencing; WGS, whole-genome sequencing.

Genes are listed under each data source when that source achieved the highest concordance with WGS analytical reference calls. If multiple data sources showed equal performance for a gene, the gene is listed under each corresponding source. Bold text indicates genes for which the integrated call set improved performance over both WES and imputed array alone.

### Gene- and platform-specific insights

Gene-level performance reflected the expected strengths of each platform. WES performed well for genes in which star-allele definitions are driven primarily by coding variation, achieving high concordance for loci such as *CYP2C9* (99.4%), *CYP3A7* (100.0%), *TPMT* (99.4%), and *NUDT15* (98.3%). In contrast, the imputed array substantially outperformed WES for several genes with important non-coding or poorly captured variants, including *VKORC1* (98.8% vs. 12.6%), *CYP2E1* (96.4% vs. 50.1%), *SLCO1B1* (98.1% vs. 30.0%), *IFNL3* (99.9% vs. 47.9%), and *CYP2C19* (97.8% vs. 64.9%). These findings suggest that array-based data more effectively capture regulatory and intronic variants that are not well represented in exome sequencing.

Across 47,136 sample–gene comparisons with a callable WGS analytical reference for each platform, Aldy reported multiple equally likely diplotype solutions for 1,125 WES calls, 2,295 imputed-array calls, and 2,304 integrated calls. Of these, 819, 1,650, and 1,659, respectively, were considered concordant under the primary any-match rule. Reclassifying all multi-solution calls as discordant reduced platform-wide concordance by 1.74, 3.50, and 3.52 percentage points for WES, imputed array, and the integrated call set, respectively. However, the effect was highly gene-specific: concordance was unchanged for 28 of 33 WES genes and 20 of 33 genes for both the imputed array and integrated call set, whereas gene-level reductions ranged from 0 to 28.90, 27.10, and 26.97 percentage points, respectively. The largest reductions were observed for *NAT2*, *CYP2E1*, and *CYP4F2*, with additional effects at *VKORC1*, *CYP3A43*, *DPYD*, and *CYP1A1*. Full gene-level results are provided in Supplementary Table S5.

### Integration benefits and challenging loci

The integrated call set improved or maintained diplotype concordance relative to the better-performing single platform for 29 of the 33 genes; the four exceptions (*CYP2D6*, *CYP3A43*, *GSTP1*, and *SLCO1B1*) showed lower concordance after integration. The integrated call set provided the greatest benefit for genes in which WES and imputed array data contributed complementary information. Improvement was evident for genes such as *CYP3A4* (WES: 92.3%, Array: 98.6%, Integrated: 99.9%), *CYP1A1* (71.9%, 97.9%, 99.5%), *NAT1* (49.7%, 93.0%, 93.4%), and *DPYD* (95.7%, 94.8%, 99.0%). *VKORC1* showed a similar pattern, with the integrated call set slightly improving upon the already strong array-based performance (WES: 12.6%, Array: 98.8%, Integrated: 99.0%), suggesting that most of the gain was driven by recovery of non-coding variation captured by the array.

The benefit of integration was not uniform across loci. For *UGT1A1*, concordance remained low and was unchanged relative to the array alone (WES: 26.6%; Array and Integrated: 44.2%). The Sankey diagram showed that several WGS-defined configurations involving *28, *60, *80, and *93 were represented by simpler diplotypes in the array and integrated call sets, indicating limited resolution of compound heterozygous configurations (Supplementary Figure S1A). For *SLCO1B1*, integration resulted in slightly lower concordance than the array alone (97.2% vs. 98.1%).

Several other loci remained difficult to resolve regardless of non-WGS platform. *GSTM1* showed only 18.2% concordance for WES, the imputed array, and the integrated call set. Because 710 participants lacked a callable WGS *GSTM1* reference owing to low coverage, these estimates should be interpreted cautiously.

For *CYP2B6*, discordance was largely attributable to the absence of a key haplotype-defining variant from the analysis call sets. The variant rs2279343, which distinguishes *6 from *9 and *4 from *1, was present in the source WES VCF but was excluded because genotype missingness was 31.86% (462/1,450), exceeding the 5% variant-level threshold. It was also absent from the imputed-array call set and therefore from the integrated call set. In contrast, rs3745274, which is shared by *6 and *9, was present in all three non-WGS datasets. Consequently, WGS-defined *6 diplotypes were frequently represented as *9 and WGS-defined *4 diplotypes as *1, indicating that integration could not compensate for the absence of the distinguishing variant.

*CYP2F1* also showed consistently low concordance (WES: 34.9%, imputed array: 40.1%, integrated: 40.1%), with the principal discordant WGS diplotypes involving *2, *3, and *5. Examination of allele-defining variants showed complementary but incomplete coverage across the non-WGS datasets (Supplementary Table S6). Among the nine variants defining *2.001 in the Aldy v4.6 allele-definition file, four were identified in WES, seven in the imputed array, and eight in the integrated call set; rs75405062 was absent from all three. The shared *2/*3 variant rs305978 was identified in WES but not in the array, whereas rs35107873, rs34903717, rs2287940, and rs305974 were identified in the array but not in WES. Selected variants defining *5 and *5.002 were also absent from the examined non-WGS datasets. These findings indicate that incomplete variant availability may contribute to *2-, *3-, and *5-related discordance.

However, synthetic VCF testing showed that variant availability did not fully explain the failure to recover *CYP2F1* *2. When all nine *2.001-defining variants, including seven SNVs and two indels, were encoded as heterozygous alternate alleles, Aldy v4.6 returned *1/*1+rs305974+rs75405062 rather than *1/*2. The same result was obtained when the variants were phased together. An indels-only VCF produced *1/*1, identical to the negative control, indicating that rs34903717 and rs3833221 were not incorporated into the *2 assignment under the tested VCF-mode conditions (Supplementary Table S7). Thus, failure to recover *2 appeared to reflect allele reconstruction under the tested Aldy v4.6 VCF workflow in addition to incomplete defining-variant coverage.

### Aldy-reported *CYP2D6* structural configurations and platform concordance

Among 1,450 participants, 1,446 had a callable *CYP2D6* WGS analytical-reference diplotype, whereas four did not because Aldy failed to produce a final phased diplotype despite copy-number evidence at high read depth. Among the 1,446 callable participants, 344 (23.8%) had at least one explicit Aldy-reported structural indicator and 1,102 (76.2%) did not. The latter group was defined by the absence of explicit structural notation rather than by an independent copy-number measurement. It may therefore include true two-copy genotypes as well as copy-number or structural configurations that were not detected, represented, or explicitly annotated in the retained Aldy output; accordingly, it was not interpreted as having normal copy number.

*CYP2D6* *5 is defined by PharmVar as a full-gene deletion; accordingly, *5 in the retained Aldy WGS output was classified here as deletion notation (Pharmacogene Variation Consortium, 2026). This designation reflects the Aldy-reported star allele rather than an independently validated copy-number measurement, and absence of *5 notation does not exclude a deletion or establish normal total copy number.

Using the mutually exclusive participant-level hierarchy, 159 participants (11.0%) had a hybrid- or conversion-associated allele, 8 (0.6%) had another mixed tandem configuration, 5 (0.3%) had same-allele multiplication, 69 (4.8%) had same-allele duplication, 103 (7.1%) had *5 deletion notation without a higher-priority structural indicator, and 1,102 (76.2%) had no explicit structural indicator. Among the 159 participants in the hybrid- or conversion-associated category, 82 (51.6%) had *36-associated structural notation and 77 (48.4%) had *68-associated structural notation; none had both. Structural indicators were evaluated across all retained WGS solutions using an any-solution rule: a participant was assigned to the explicit-structural stratum if any retained WGS solution contained a structural indicator. Thus, this rule was deliberately inclusive with respect to the 344-participant explicit-structural stratum. Of the 1,446 callable WGS results, 1,441 (99.7%) had a single retained solution and 5 (0.3%) had multiple retained solutions. Detailed observed motifs and participant-level categories are reported in Supplementary Tables S8, S9.

Under the primary any-match rule, concordance with the WGS analytical reference was 51.9% for WES, 54.6% for the imputed array, and 51.8% for the integrated call set. Multi-solution calls occurred in 180 WES results (12.4%), 1 imputed-array result (0.1%), and 2 integrated results (0.1%); the remaining 87.6%, 99.9%, and 99.9%, respectively, contained a single candidate diplotype. Under the strict rule, which classified all multi-solution calls as discordant, concordance was 43.2%, 54.6%, and 51.8%, respectively (Table 3). Thus, the any-match rule materially affected WES *CYP2D6* concordance but not the array or integrated estimates. The primary any-match rule measures whether the analytical-reference diplotype was recoverable among the returned candidates and does not imply that a multi-solution result is unambiguous or clinically resolved. For none of the 344 participants with an explicit structural indicator did any non-WGS platform reproduce the configuration reported by the WGS-based Aldy analysis, whereas concordance under the primary any-match rule among the 1,102 participants without explicit structural notation was 68.1%, 71.7%, and 68.0% for WES, imputed array, and integrated calls, respectively (Supplementary Table S9). Explicit structural notation in the analytical reference was therefore present in a substantial, but incomplete, subset of discordant cases. All *CYP2D6* concordance estimates reported here quantify agreement with a single-caller WGS analytical reference and are not estimates of clinical *CYP2D6* typing accuracy.

**TABLE 3:** *CYP2D6* concordance under primary any-match and strict multi-solution rules.

Measure	WES	Imputed array	Integrated
Evaluable, n	1,446	1,446	1,446
Any-match concordance, n (%)	750 (51.9)	790 (54.6)	749 (51.8)
Strict concordance, n (%)	625 (43.2)	790 (54.6)	749 (51.8)
Multi-solution calls, n (%)	180 (12.4)	1 (0.1)	2 (0.1)
Single-candidate calls, n (%)	1,266 (87.6)	1,445 (99.9)	1,444 (99.9)
Strict: explicit structural indicator, n/N (%)	0/344 (0.0)	0/344 (0.0)	0/344 (0.0)
Strict: no explicit structural indicator, n/N (%)	625/1,102 (56.7)	790/1,102 (71.7)	749/1,102 (68.0)

Evaluable participants had a callable *CYP2D6* WGS analytical reference. Under the primary any-match rule, a test call was concordant if any retained candidate diplotype matched a retained WGS solution; under the strict rule, all test calls with multiple equally likely solutions were classified as discordant. Structural-notation strata were defined from the WGS analytical reference as described in Supplementary Table S9.

The four participants without a callable *CYP2D6* WGS analytical reference could not be evaluated for exact concordance or assigned to a structural stratum. Treating all four as discordant in a conservative sensitivity analysis changed aggregate concordance by at most 0.15 percentage points (WES, 51.7%; imputed array, 54.5%; integrated, 51.7%). Supplementary Tables S8, S9 summarize structural notation present in the Aldy-derived WGS analytical reference and do not establish the accuracy of *CYP2D6* copy-number or structural inference in the absence of independent validation.

To assess whether coordinate conversion contributed to the *CYP2D6* findings, we re-examined the array LiftOver procedure. Five of 978 *CYP2D6* array variants (0.51%) failed GRCh37-to-GRCh38 mapping and were annotated by UCSC LiftOver as “Deleted in new.” Only one, rs28735595, was present in the Aldy definition of *CYP2D6* *132.001. After correcting the LiftOver handling, regenerating the array and integrated VCFs, and repeating Aldy-based calling, the integrated call set produced identical sample-level diplotype solutions and unchanged WGS concordance of 51.8%.

### Ancestry-stratified WGS-concordance pattern

Concordance of star-allele calls from WES, array, and integrated datasets against WGS varied across ancestry groups (Figure 2). Several genes—including *CYP3A4* and *DPYD*—showed consistently high concordance across all populations (*CYP3A4*: WES 92.3%–95.7%, Array 97.3%–99.6%, Integrated 99.3%–100.0%; *DPYD*: WES 95.0%–96.8%, Array 94.1%–95.4%, Integrated 98.6%–99.3%). In contrast, other genes exhibited population-specific differences in platform performance. For example, *CYP2C19* showed uniformly high concordance in the integrated and array-based call sets across ancestries (≥97.9%), but markedly lower concordance from WES in European (60.1%), African (63.3%), and South Asian (67.3%) populations. This reflects WES’s inability to detect *17, a common non-coding promoter variant (rs12248560) not captured by WES (Gaedigk et al., 2022). East Asians showed much higher WES concordance for *CYP2C19* (96.0%), likely due to the low prevalence of *17 in this population (Supplementary Figure S1B). These patterns indicate that population-specific variant frequencies contributed to ancestry differences in platform concordance, particularly when key alleles lay outside the exome.

**FIGURE 2:**
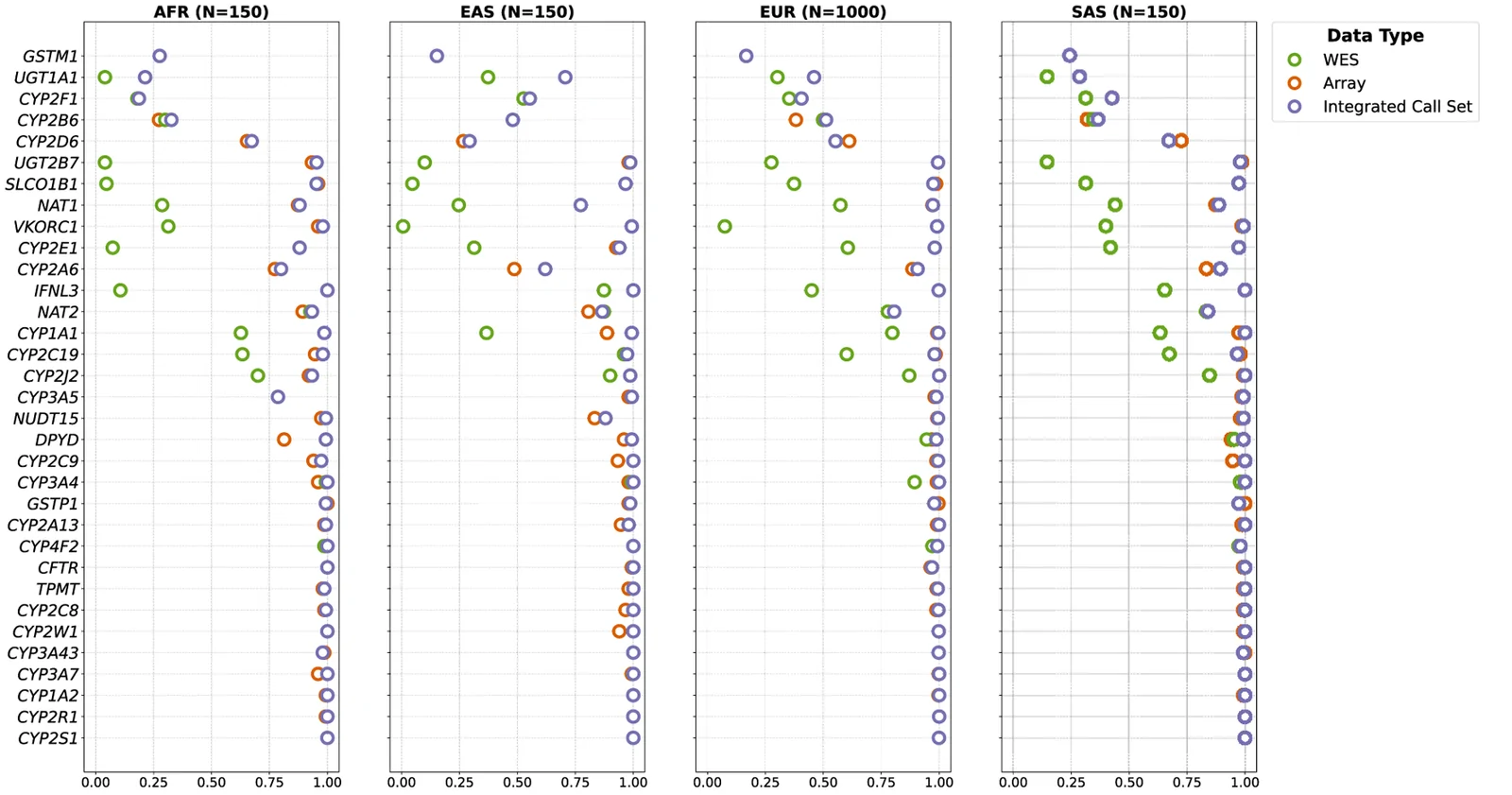
Gene-wise Concordance with WGS Across Data Types and Populations Dot plot showing concordance with WGS analytical reference star-allele calls across four populations—African, East Asian, European, and South Asian—for 33 pharmacogenes. Concordance was calculated among individuals with a callable WGS analytical reference for each gene, with gene- and ancestry-specific sample sizes shown in each panel. Each panel compares WES, imputed array, and the integrated call set. Gene order is fixed across panels for direct comparison.

Beyond *CYP2C19*, other genes also demonstrated ancestry-related differences. For instance, *SLCO1B1* showed high array-based concordance across ancestries (≥98.0%), but WES performance was substantially lower, particularly in non-European populations (AFR: 14.1%; SAS: 21.0%). Similarly, *CYP2D6*, a gene with complex structural variation, showed substantial ancestry variation in concordance across platforms (WES: 26.7%–65.8%; Array: 24.0%–71.1%; Integrated: 26.7%–65.8%), with the lowest agreement observed in EAS participants and highest in SAS participants.

### Ancestry-stratified star-allele frequencies

Star-allele haplotype frequencies were summarized from Aldy-derived WGS calls and varied across ancestry groups for 33 pharmacogenes (Supplementary Figure S1B). Across the broader set of genes examined, the ancestry-stratified haplotype patterns were generally consistent with previously reported population allele distributions and clinically established functional annotations (Twesigomwe et al., 2020; Marez et al., 1997; Liau et al., 2019; Gaedigk et al., 1991; Gaedigk et al., 2022; Blaisdell et al., 2004; King et al., 2004).

### Characterizing discordant diplotype calls with union-of-FAIL sankey diagrams

To examine the composition of discordant diplotype calls beyond overall concordance, we focused on a per-gene subset of individuals with a callable WGS analytical reference in whom at least one non-WGS platform—WES, imputed array, or the integrated call set—produced a diplotype that did not match the WGS analytical reference (“union-of-FAIL”). Within this subset, we quantified platform-specific discordance counts and the net change in concordant calls after integration relative to WES and imputed array (Table 4), and visualized the underlying patterns of gain and loss using a multi-panel union-of-FAIL framework (Figure 3; Supplementary Figure S1).

**TABLE 4:** Platform-specific discordance and net change by WES–array integration within union-of-FAIL samples, stratified by gene.

Gene	Union-of-FAIL	WES discordant	Array discordant	Integrated call set discordant	Net change vs. WES	Net change vs. Array
*CFTR*	39	31	39	31	0	8
*CYP1A1*	413	408	31	7	401	24
*CYP1A2*	5	0	5	0	0	5
*CYP2A13*	23	5	21	5	0	16
*CYP2A6*	277	205	260	205	0	55
*CYP2B6*	925	782	907	762	20	145
*CYP2C19*	525	509	32	32	477	0
*CYP2C8*	22	4	20	4	0	16
*CYP2C9*	40	9	39	9	0	30
*CYP2D6*	810	696	656	697	−1	−41
*CYP2E1*	726	723	52	50	673	2
*CYP2F1*	946	944	869	869	75	0
*CYP2J2*	219	212	15	12	200	3
*CYP2R1*	1	0	1	0	0	1
*CYP2S1*	1	1	1	1	0	0
*CYP2W1*	14	2	12	2	0	10
*CYP3A4*	130	111	21	1	110	20
*CYP3A43*	9	6	4	6	0	−2
*CYP3A5*	58	46	57	46	0	11
*CYP3A7*	9	0	9	0	0	9
*CYP4F2*	40	35	14	11	24	3
*DPYD*	130	63	75	15	48	60
*GSTM1*	605	605	605	605	0	0
*GSTP1*	30	28	6	28	0	−22
*IFNL3*	755	755	2	2	753	0
*NAT1*	739	730	101	96	634	5
*NAT2*	310	258	247	230	28	17
*NUDT15*	40	25	40	25	0	15
*SLCO1B1*	1017	1015	28	41	974	−13
*TPMT*	24	8	18	8	0	10
*UGT1A1*	1065	1064	809	809	255	0
*UGT2B7*	1133	1131	20	17	1114	3
*VKORC1*	1269	1267	18	14	1253	4

Union-of-FAIL samples were individuals with a callable WGS analytical reference whose diplotype from at least one non-WGS platform did not match any retained WGS solution. Discordant indicates platform-specific mismatches. Net change was calculated as platform discordance minus integrated-call-set discordance; positive and negative values indicate net gain and loss, respectively. Samples without a callable WGS result were excluded.

**FIGURE 3:**
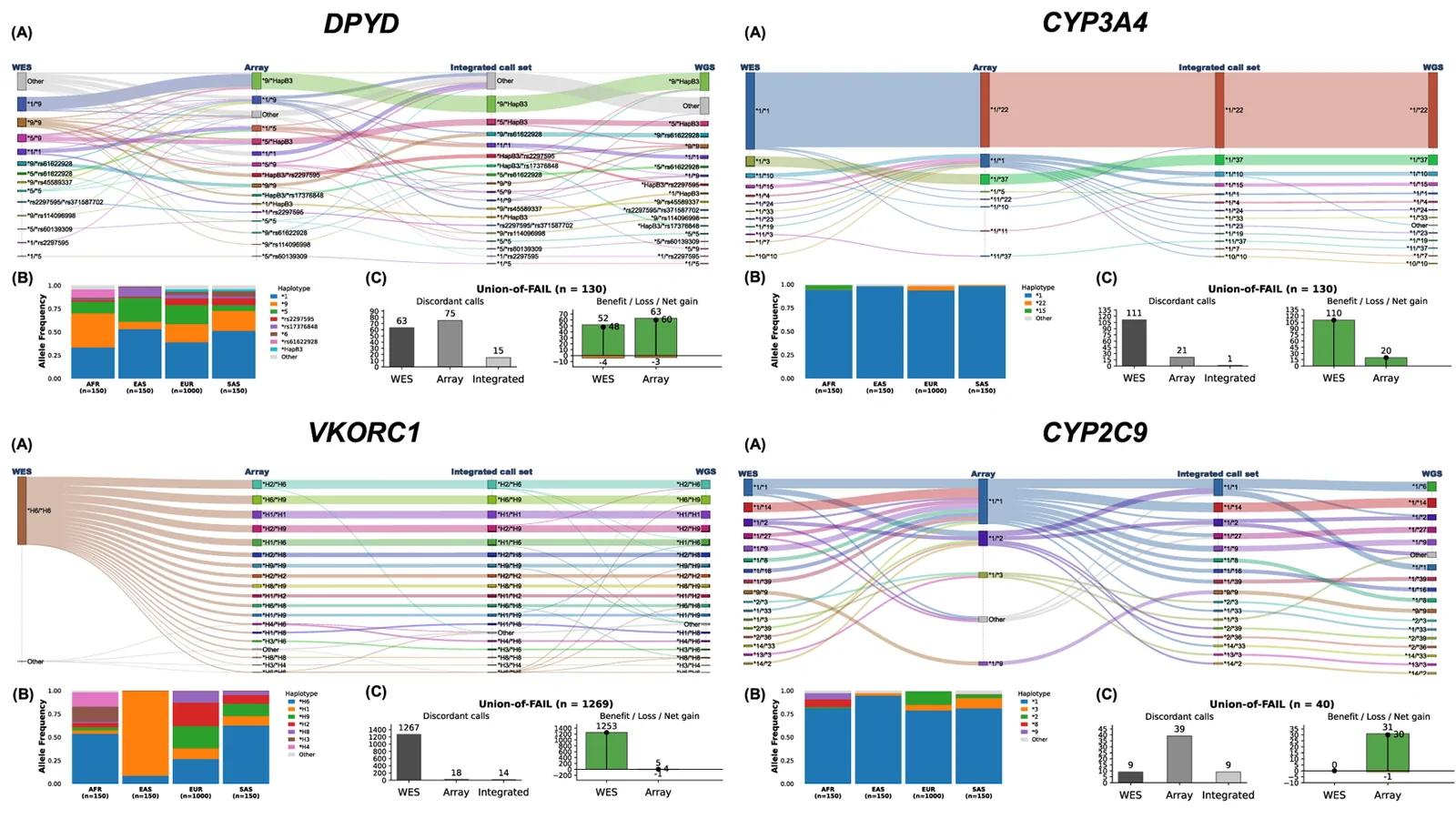
Union-of-FAIL analysis reveals representative platform-specific discordance patterns and net effects of WES–array integration across four pharmacogenes (*DPYD*, *CYP3A4*, *VKORC1*, and *CYP2C9*) For each gene, union-of-FAIL samples were defined as individuals for whom at least one non-WGS platform—WES, imputed array, or the integrated call set—produced a diplotype that did not match the WGS analytical reference. Four representative genes are shown in a 2 × 2 arrangement. Within each gene panel set, panel **(A)** shows a Sankey diagram summarizing diplotype flows across WES, imputed array, the integrated call set, and WGS, with link widths proportional to the number of individuals transitioning between diplotypes across adjacent stages. Panel **(B)** shows WGS analytical reference star-allele frequency distributions across ancestry groups, providing population context for the ancestry-specific composition of discordant calls observed in the union-of-FAIL subset. Panel **(C)** summarizes platform-specific discordance within the union-of-FAIL subset and the number of discordant calls rescued by the integrated call set relative to WES or imputed array. Corresponding plots for all pharmacogenes are provided in Supplementary Figure S1.

Union-of-FAIL counts ranged from 1 for *CYP2R1* and *CYP2S1* to 1,269 for *VKORC1*, followed by 1,133 for *UGT2B7*. The dominant source of discordance differed by gene: WES contributed more discordant calls than imputed array for *IFNL3*, *SLCO1B1*, *CYP2E1*, *NAT1*, and *CYP2C19*, whereas imputed array contributed more for *CYP2A6*, *CYP3A5*, and *NUDT15*. Integration yielded the largest net gains over WES for *VKORC1* (+1,253), *UGT2B7* (+1,114), *SLCO1B1* (+974), *IFNL3* (+753), *CYP2E1* (+673), and *NAT1* (+634), and over imputed array for *CYP2B6* (+145), *DPYD* (+60), *CYP2A6* (+55), and *CYP2C9* (+30). In contrast, net losses relative to imputed array were observed for *CYP2D6* (−41), *GSTP1* (−22), *SLCO1B1* (−13), and *CYP3A43* (−2).

Representative examples in Figure 3 reflect these distinct patterns. *DPYD* showed net gain in the integrated call set relative to both WES and imputed array, whereas *VKORC1* showed large gain primarily relative to WES. *CYP3A4* showed very high concordance after integration (only 1 discordant call). Although the improvement over WES was largely driven by array-supported correction, the Sankey diagram further highlights that the integrated call set preserves the diversity of rare star-allele calls originally captured by WES. In contrast, *CYP2C9* illustrated a locus in which WES already showed high concordance, whereas imputed array showed substantial discordance; in this case, integration largely restored the WES-consistent diplotype pattern.

## Discussion

In this study of 1,450 multi-ancestry UKBB participants, we evaluated pharmacogenetic diplotype concordance from WES, imputed genotyping arrays, and an integrated call set using Aldy-derived WGS calls as the analytical reference across 33 pharmacogenes. Integration improved or maintained diplotype concordance for most genes compared with WES or array alone, supporting the utility of combining complementary variant evidence for population-scale pharmacogenetic research.

Our study builds on prior work such as PGxPOP, which documented cross-platform differences in UK Biobank but did not compare calls with an external high-resolution analytical reference (McInnes et al., 2021). By anchoring the evaluation to an Aldy-derived WGS analytical reference, we characterized analytical concordance rather than clinical typing accuracy and distinguished loci where discordance likely reflected platform limitations from loci where the non-WGS calls agreed with the analytical reference.

At the gene level, performance patterns were consistent with the expected strengths of each data source: WES generally performed best for loci driven primarily by coding variation, whereas imputed arrays performed best for genes where key determinants are non-coding or regulatory. Integration was most beneficial for genes where both classes of variants contribute and where each single platform incompletely captures the haplotype-defining variation. Conversely, for some genes, a single non-WGS platform may already achieve high concordance with WGS, leaving little additional benefit from integration. *SLCO1B1* illustrates this pattern: imputed array alone reached near-complete concordance with WGS (98.1%), whereas WES performed substantially worse (30.0%), and the integrated call set fell slightly below array alone (97.2%). This suggests that, at such loci, integration mainly compensates for the weaker platform rather than surpassing an already strong single-platform source. Separately, several loci showed persistently low concordance across all non-WGS inputs, consistent with variant architectures dominated by structural variation and copy-number complexity that are not well represented in VCF-based genotyping.

Beyond summary concordance, “union-of-FAIL” profiling using Sankey diagrams clarified how integration reduces discordance rather than merely increasing a global percentage. Because many individuals carry common diplotypes, overall concordance can obscure rescue behavior; focusing on samples that were discordant in at least one non-WGS platform highlighted that integration tends to selectively leverage the more informative platform for a given locus, reducing discordant calls without simply averaging inputs.

The expanded union-of-FAIL framework also showed that the impact of integration cannot always be fully summarized by the number of rescued calls alone. For example, in *CYP3A4*, the numerical summaries indicated substantial rescue after integration, but the Sankey diagrams further showed that the integrated call set preserved the diversity of rare star-allele calls captured by WES while also recovering additional WGS-matching calls (Figure 3). This illustrates that integration may improve performance not only by reducing discordance, but also by retaining diplotype resolution that would be less apparent from concordance percentages alone. Similarly, the ancestry-stratified WGS star-allele frequency panels highlighted that discordance patterns were shaped in part by population-specific allele composition, helping explain why the same platform could perform differently across genes with distinct underlying haplotype structures.

From a translational perspective, these results suggest that the utility of existing WES and genotyping-array resources for pharmacogenetic research is gene-dependent. For genes such as *VKORC1* and *SLCO1B1*, array-derived or integrated calls achieved high concordance with WGS, supporting their use in large-scale retrospective PGx analyses when WGS is unavailable. However, the any-match rule is appropriate for benchmarking analytical performance rather than clinical implementation, because ambiguous diplotype calls may correspond to different predicted phenotypes. Accordingly, the high concordance observed for many pharmacogenes should not be interpreted as indicating that integrated WES–array call sets can universally replace dedicated clinical pharmacogenetic testing. Loci with persistent diplotype ambiguity or complex variant architectures may therefore require orthogonal confirmation or specialized pharmacogenetic assays before clinical implementation.

For *CYP2D6*, strict handling reduced WES concordance from 51.9% to 43.2%, emphasizing that primary any-match concordance reflects analytical recoverability rather than unambiguous diplotype resolution. None of the structural configurations reported by the WGS-based Aldy analysis were reproduced by the evaluated non-WGS workflows. This finding does not by itself distinguish among missing structural information in the non-WGS datasets, uncertainty or error in the WGS-based Aldy output, limitations of VCF representation, star-allele definitions or parsing, and phasing or allele-reconstruction error; these possibilities cannot be separated without an orthogonal structural assay. Analytical concordance therefore does not establish activity-score or metabolizer-phenotype validity, and the evaluated VCF-based workflows should not substitute for validated CNV- and structure-aware *CYP2D6* testing when clinical phenotype assignment is required.

Several limitations should be considered. First, the WGS analytical reference was generated using a single computational caller and was not orthogonally validated. This limitation is particularly relevant to structurally complex loci for which the retained Aldy notation should not be interpreted as independently verified copy number, breakpoint structure, or phase. Second, all calls were generated using Aldy v4.6 and current database annotations; ongoing updates to star-allele definitions and functional mappings may change interpretations over time. Third, although we included four major ancestry groups, non-European sample sizes were smaller, limiting precision for rare diplotypes and ancestry-stratified estimates. Fourth, the findings reflect the properties of United Kingdom Biobank sequencing, imputation, and ancestry composition, and may differ in cohorts generated with other platforms or reference panels. Finally, although the WGS analytical reference provided a useful analytical benchmark for platform comparison, this study did not constitute clinical validation against orthogonal pharmacogenetic assays; therefore, clinical implementation of integrated calls would require additional validation, particularly for high-impact genes and loci with complex variation.

We developed PGxIntegrator, an end-to-end workflow for harmonization, liftover, WES–array integration, and parallelized star-allele calling, designed to support scalable population-level PGx analyses and straightforward reprocessing as reference resources and allele definitions are updated. Although evaluated here using WES and genotyping-array data, the framework could be extended to other variant sources, including targeted sequencing or long-read-derived call sets, provided they are represented in compatible VCF or BGEN-derived formats. The current implementation retains only the genotype field during integration. Although phased GT separators may remain represented, phase-set annotations and the phase-block information required to link variants across a haplotype are not preserved. Extending the framework to preserve phase-set information, together with downstream PGx callers capable of using it, could enable phased information from long-read or other phase-aware assays to further improve diplotype resolution and represents a natural direction for future development. These gene-level distinctions are important for prioritizing when existing WES or array resources may be sufficient for pharmacogenetic research and when WGS, long-read sequencing, or SV/CNV-aware approaches remain necessary.

Overall, our results support WES–array integration as a practical strategy for scalable pharmacogenetic typing in cohorts where WGS is unavailable, while highlighting gene classes where non-WGS approaches remain limited and improved SV/CNV-aware methods are needed. For *CYP2D6* specifically, concordance reflects agreement with a single-caller WGS analytical reference rather than clinical typing accuracy. The evaluated VCF-based integration workflow did not reproduce the structural configurations reported by the WGS-based Aldy analysis, highlighting the need for CNV- and structure-aware approaches when accurate *CYP2D6* structural resolution is required.

## Data Availability

The data analyzed in this study are available through the UK Biobank Research Analysis Platform upon application and approval by UK Biobank. The data were accessed under UK Biobank application number 45227 and are subject to UK Biobank data-access policies. The PGxIntegrator pipeline is publicly available at https://github.com/achl1990/PGxIntegrator.
