# Supplementary material for: Integrating whole-exome and genotyping array data for pharmacogenetic typing: a comparative evaluation with whole-genome sequencing in UK Biobank
Source: Front Pharmacol. 2026 Oct 26;17:1892708. doi: 10.3389/fphar.2026.1892708 (PMC13745935; doi:10.3389/fphar.2026.1892708)
Supplement: Supplementary file 1 [file Supplementaryfile1.docx]

**Supplementary Methods**

**CYP2D6 diplotype normalization and exact concordance matching**

For concordance comparisons, Aldy Major-field diplotype solutions were normalized identically for the WGS analytical reference and WES, imputed-array, and integrated call sets. Whitespace and appended novel-variant annotations matching +rs<number> were removed, while structural and suballelic notation, including “+”-joined tandem components, *5, .ALDY and lettered suffixes, and :2 notation, was retained. The order of the two “/”-separated haplotypes was canonicalized so that, for example, *1/*2 and *2/*1 were equivalent; the internal order of “+”-joined components was preserved. For samples with multiple equally likely solutions, each complete diplotype was normalized independently, and concordance was assigned when at least one normalized test solution exactly matched one normalized WGS solution. No component-level partial matching was performed.

**Pseudocode**

Normalize(diplotype):

1. use the Aldy Major field
2. remove whitespace and +rs<number> annotations
3. preserve structural and suballelic notation
4. split the diplotype at “/”
5. sort the two haplotypes and rejoin with “/”
6. do not reorder components within “+”

Concordant(test, WGS):

1. normalize all retained test solutions
2. normalize all retained WGS solutions
3. return concordant if the two normalized solution sets have a non-empty intersection

**Supplementary Tables**

**Supplementary Table S1. Summary of demographic characteristics by ancestry group**

| **Ethnicity** | **N** | **Mean Age (±SD)** | **Female (%)** |
| --- | --- | --- | --- |
| African (AFR) | 150 | 52.4 ± 8.4 | 43.3 |
| East Asian (EAS) | 150 | 53.4 ± 7.8 | 62.0 |
| European (EUR) | 1000 | 56.5 ± 8.0 | 57.4 |
| South Asian (SAS) | 150 | 53.7 ± 8.5 | 48.0 |

N, number of individuals; SD, standard deviation. Non-European groups were down-sampled due to limited availability in the UK Biobank

**Supplementary Table S2. Whole-exome sequencing depth summary by pharmacogene.** For each gene, total SNP sites (n) indicates the number of SNP loci observed in the WES VCF. Mean depth (DP) denotes the average genotype-level read depth (VCF DP field) across these SNP sites after genotype-level QC excluding genotypes with DP < 10. These gene-level depth summaries provide coverage context for interpreting WES-based star-allele typing performance

| **Gene** | **Total SNP sites (n)** | **Mean Depth (DP)** |
| --- | --- | --- |
| *CFTR* | 3671 | 17.63 |
| *CYP1A1* | 769 | 18.63 |
| *CYP1A2* | 875 | 18.37 |
| *CYP2A13* | 1260 | 25.62 |
| *CYP2A6* | 1282 | 31.42 |
| *CYP2B6* | 1026 | 18.34 |
| *CYP2C19* | 1095 | 18.42 |
| *CYP2C8* | 875 | 18.37 |
| *CYP2C9* | 1197 | 18.48 |
| *CYP2D6* | 1514 | 26.03 |
| *CYP2E1* | 1820 | 19.71 |
| *CYP2F1* | 918 | 19.00 |
| *CYP2J2* | 769 | 17.22 |
| *CYP2R1* | 1280 | 20.24 |
| *CYP2S1* | 1063 | 24.54 |
| *CYP2W1* | 1619 | 20.67 |
| *CYP3A43* | 886 | 17.46 |
| *CYP3A4* | 819 | 18.07 |
| *CYP3A5* | 1599 | 18.59 |
| *CYP3A7* | 1112 | 19.85 |
| *CYP4F2* | 1225 | 27.74 |
| *DPYD* | 2273 | 20.12 |
| *GSTM1* | 750 | 19.93 |
| *GSTP1* | 362 | 19.45 |
| *IFNL3* | 1161 | 22.02 |
| *NAT1* | 451 | 19.13 |
| *NAT2* | 400 | 28.05 |
| *NUDT15* | 326 | 17.63 |
| *SLCO1B1* | 783 | 17.35 |
| *TPMT* | 925 | 19.39 |
| *UGT1A1* | 1057 | 40.90 |
| *UGT2B7* | 852 | 22.19 |
| *VKORC1* | 2680 | 25.66 |

**Supplementary Table S3 Platform-specific unique variant positions by gene.** The number of variant positions uniquely detected by either imputed array (array-only) or WES (WES-only) is shown for each gene. This highlights the complementary strengths of each platform in capturing different sets of variants. Gene lengths (in base pairs) are included to provide context for variant density and coverage differences.

| **Gene** | **WES-only** | **Array-only** | **Gene Length (bp)** |
| --- | --- | --- | --- |
| ***CYP1* Family** | | | |
| *CYP1A1* | 680 | 735 | 5,995 |
| *CYP1A2* | 801 | 795 | 7,763 |
| ***CYP2* Family** | | | |
| *CYP2A6* | 980 | 823 | 6,907 |
| *CYP2A13* | 1,012 | 941 | 7,745 |
| *CYP2B6* | 949 | 2,281 | 27,117 |
| *CYP2C8* | 966 | 1,647 | 32,726 |
| *CYP2C9* | 1,230 | 2,771 | 51,434 |
| *CYP2C19* | 1,071 | 7,663 | 92,867 |
| *CYP2D6* | 1111 | 774 | 4,367 |
| *CYP2E1* | 1,943 | 2,012 | 40,815 |
| *CYP2F1* | 918 | 1,247 | 13,950 |
| *CYP2J2* | 916 | 1,589 | 33,466 |
| *CYP2R1* | 1,359 | 869 | 14,792 |
| *CYP2S1* | 896 | 1,202 | 14,330 |
| *CYP2W1* | 1,424 | 1,027 | 6,460 |
| ***CYP3* Family** | | | |
| *CYP3A4* | 979 | 1,294 | 27,289 |
| *CYP3A5* | 1,773 | 1,366 | 31,803 |
| *CYP3A7* | 1,089 | 1,492 | 30,161 |
| *CYP3A43* | 1,117 | 1,709 | 38,081 |
| ***CYP4* Family** | | | |
| *CYP4F2* | 1,013 | 1,358 | 20,055 |
| **Glutathione S-Transferases** | | | |
| *GSTM1* | 1,204 | 1,009 | 21,226 |
| *GSTP1* | 522 | 776 | 2,915 |
| **UDP-Glucuronosyltransferases** | | | |
| *UGT1A1* | 965 | 1,052 | 13,031 |
| *UGT2B7* | 789 | 3,052 | 61,625 |
| **N-Acetyltransferases** | | | |
| *NAT1* | 517 | 3,322 | 53,213 |
| *NAT2* | 345 | 1,285 | 9,937 |
| **Others** | | | |
| *CFTR* | 5,095 | 7,075 | 428,852 |
| *DPYD* | 2,688 | 27,343 | 917,258 |
| *IFNL3* | 1,025 | 659 | 1,796 |
| *NUDT15* | 357 | 1,039 | 9,496 |
| *SLCO1B1* | 1,277 | 4,381 | 108,603 |
| *TPMT* | 1,204 | 1,432 | 26,767 |
| *VKORC1* | 2,463 | 454 | 5,139 |

**Supplementary Table S4 Gene-level annotation sources and support status in Aldy v4.6**

| **Gene** | **Annotation Source** |
| --- | --- |
| *CYP2D6* | PharmVar 5.2.3 |
| *CYP2A6* | PharmVar 5.2.3 |
| *CYP2B6* | PharmVar 5.2.3 |
| *CYP1A1* | PharmGKB (Dec 2014) and Pharmacoscan R9 |
| *CYP1A2* | PharmGKB (Mar 2014) and Pharmacoscan R9 |
| *CYP2A13* | PharmVar 5.2.3 |
| *CYP2C19* | PharmVar 5.2.3 |
| *CYP2C8* | PharmVar 5.2.3 |
| *CYP2C9* | PharmVar 5.2.3 |
| *CYP2E1* | PharmGKB (Nov 2013) |
| *CYP2F1* | PharmVar 5.2.3 |
| *CYP2J2* | PharmVar 5.2.3 |
| *CYP2R1* | PharmVar 5.2.3 |
| *CYP2S1* | PharmVar 5.2.3 |
| *CYP2W1* | PharmVar 5.2.3 |
| *CYP3A43* | PharmVar 5.2.3 |
| *CYP3A4* | PharmVar 5.2.3 |
| *CYP3A5* | PharmVar 5.2.3 |
| *CYP3A7* | PharmVar 5.2.3 |
| *CYP4F2* | PharmVar 5.2.3 |
| *CFTR* | PharmGKB (Jun 2020) and Pharmacoscan R9 |
| *DPYD* | PharmVar 5.2.3 |
| *GSTM1* | Pharmacoscan R9 |
| *GSTP1* | Pharmacoscan R9 |
| *IFNL3* | PharmGKB and Pharmacoscan R9 |
| *NAT1* | PharmGKB (Mar 2014) and Pharmacoscan R9 |
| *NAT2* | PharmGKB (Mar 2014) and Pharmacoscan R9 |
| *NUDT15* | PharmVar 5.2.3 |
| *SLCO1B1* | PharmVar 5.2.3 |
| *TPMT* | PharmGKB (Jun 2020) and Pharmacoscan R9 |
| *UGT1A1* | PharmGKB (Feb 2020) and Pharmacoscan R9 |
| *UGT2B7* | pharmacogenomics.pha.ulaval.ca (Apr 2015) / Pharmacoscan R9 |
| *VKORC1* | PharmGKB (Jan 2021) and Pharmacoscan R9 |

**Supplementary Table S5. Gene-level sensitivity analysis of Aldy multi-solution calls across WES, imputed-array, and integrated call sets.** All 33 pharmacogenes are shown for each platform. " Evaluable, n” denotes comparisons with a callable WGS analytical reference. "Any-match concord., n" denotes multi-solution calls considered concordant because at least one candidate diplotype matched WGS. In the strict analysis, all multi-solution calls were classified as discordant. "Change, pp" is strict minus original concordance. Platform-wide changes were calculated across all evaluable sample–gene comparisons.

| **Platform** | **Gene** | **Evaluable, n** | **Multi-solution calls, n** | **Any-match concord., n** | **Original concord., %** | **Strict concord., %** | **Change, pp** |
| --- | --- | --- | --- | --- | --- | --- | --- |
| WES | *CFTR* | 1450 | 0 | 0 | 97.86 | 97.86 | 0.00 |
| WES | *CYP1A1* | 1450 | 0 | 0 | 71.86 | 71.86 | 0.00 |
| WES | *CYP1A2* | 1450 | 0 | 0 | 100.00 | 100.00 | 0.00 |
| WES | *CYP2A13* | 1450 | 0 | 0 | 99.66 | 99.66 | 0.00 |
| WES | *CYP2A6* | 1450 | 0 | 0 | 85.86 | 85.86 | 0.00 |
| WES | *CYP2B6* | 1450 | 0 | 0 | 46.07 | 46.07 | 0.00 |
| WES | *CYP2C8* | 1450 | 0 | 0 | 99.72 | 99.72 | 0.00 |
| WES | *CYP2C9* | 1450 | 0 | 0 | 99.38 | 99.38 | 0.00 |
| WES | *CYP2C19* | 1450 | 0 | 0 | 64.90 | 64.90 | 0.00 |
| WES | *CYP2D6* | 1446 | 180 | 125 | 51.87 | 43.22 | **−8.64** |
| WES | *CYP2E1* | 1450 | 0 | 0 | 50.14 | 50.14 | 0.00 |
| WES | *CYP2F1* | 1450 | 0 | 0 | 34.90 | 34.90 | 0.00 |
| WES | *CYP2J2* | 1450 | 0 | 0 | 85.38 | 85.38 | 0.00 |
| WES | *CYP2R1* | 1450 | 0 | 0 | 100.00 | 100.00 | 0.00 |
| WES | *CYP2S1* | 1450 | 0 | 0 | 99.93 | 99.93 | 0.00 |
| WES | *CYP2W1* | 1450 | 0 | 0 | 99.86 | 99.86 | 0.00 |
| WES | *CYP3A4* | 1450 | 0 | 0 | 92.34 | 92.34 | 0.00 |
| WES | *CYP3A5* | 1450 | 0 | 0 | 96.83 | 96.83 | 0.00 |
| WES | *CYP3A7* | 1450 | 0 | 0 | 100.00 | 100.00 | 0.00 |
| WES | *CYP3A43* | 1450 | 101 | 101 | 99.59 | 92.62 | **-6.97** |
| WES | *CYP4F2* | 1450 | 0 | 0 | 97.59 | 97.59 | 0.00 |
| WES | *DPYD* | 1450 | 90 | 87 | 95.66 | 89.66 | **-6.00** |
| WES | *GSTM1* | 740 | 0 | 0 | 18.24 | 18.24 | 0.00 |
| WES | *GSTP1* | 1450 | 0 | 0 | 98.07 | 98.07 | 0.00 |
| WES | *IFNL3* | 1450 | 0 | 0 | 47.93 | 47.93 | 0.00 |
| WES | *NAT1* | 1450 | 48 | 0 | 49.66 | 49.66 | 0.00 |
| WES | *NAT2* | 1450 | 627 | 419 | 82.21 | 53.31 | **-28.90** |
| WES | *NUDT15* | 1450 | 0 | 0 | 98.28 | 98.28 | 0.00 |
| WES | *SLCO1B1* | 1450 | 0 | 0 | 30.00 | 30.00 | 0.00 |
| WES | *TPMT* | 1450 | 79 | 78 | 99.45 | 94.07 | **-5.38** |
| WES | *UGT1A1* | 1450 | 0 | 0 | 26.62 | 26.62 | 0.00 |
| WES | *UGT2B7* | 1450 | 0 | 0 | 22.00 | 22.00 | 0.00 |
| WES | *VKORC1* | 1450 | 0 | 0 | 12.62 | 12.62 | 0.00 |
| Array | *CFTR* | 1450 | 0 | 0 | 97.31 | 97.31 | 0.00 |
| Array | *CYP1A1* | 1450 | 98 | 94 | 97.86 | 91.38 | **-6.48** |
| Array | *CYP1A2* | 1450 | 0 | 0 | 99.66 | 99.66 | 0.00 |
| Array | *CYP2A13* | 1450 | 0 | 0 | 98.55 | 98.55 | 0.00 |
| Array | *CYP2A6* | 1450 | 0 | 0 | 82.07 | 82.07 | 0.00 |
| Array | *CYP2B6* | 1450 | 0 | 0 | 37.45 | 37.45 | 0.00 |
| Array | *CYP2C8* | 1450 | 0 | 0 | 98.62 | 98.62 | 0.00 |
| Array | *CYP2C9* | 1450 | 0 | 0 | 97.31 | 97.31 | 0.00 |
| Array | *CYP2C19* | 1450 | 1 | 1 | 97.79 | 97.72 | **-0.07** |
| Array | *CYP2D6* | 1446 | 1 | 0 | 54.63 | 54.63 | 0.00 |
| Array | *CYP2E1* | 1450 | 304 | 291 | 96.41 | 76.34 | **-20.07** |
| Array | *CYP2F1* | 1450 | 23 | 0 | 40.07 | 40.07 | 0.00 |
| Array | *CYP2J2* | 1450 | 0 | 0 | 98.97 | 98.97 | 0.00 |
| Array | *CYP2R1* | 1450 | 0 | 0 | 99.93 | 99.93 | 0.00 |
| Array | *CYP2S1* | 1450 | 0 | 0 | 99.93 | 99.93 | 0.00 |
| Array | *CYP2W1* | 1450 | 0 | 0 | 99.17 | 99.17 | 0.00 |
| Array | *CYP3A4* | 1450 | 13 | 13 | 98.55 | 97.66 | **-0.9** |
| Array | *CYP3A5* | 1450 | 0 | 0 | 96.07 | 96.07 | 0.00 |
| Array | *CYP3A7* | 1450 | 0 | 0 | 99.38 | 99.38 | 0.00 |
| Array | *CYP3A43* | 1450 | 104 | 104 | 99.72 | 92.55 | **-7.17** |
| Array | *CYP4F2* | 1450 | 270 | 267 | 99.03 | 80.62 | **-18.41** |
| Array | *DPYD* | 1450 | 100 | 97 | 94.83 | 88.14 | **-6.69** |
| Array | *GSTM1* | 740 | 0 | 0 | 18.24 | 18.24 | 0.00 |
| Array | *GSTP1* | 1450 | 0 | 0 | 99.59 | 99.59 | 0.00 |
| Array | *IFNL3* | 1450 | 0 | 0 | 99.86 | 99.86 | 0.00 |
| Array | *NAT1* | 1450 | 61 | 60 | 93.03 | 88.90 | **-4.14** |
| Array | *NAT2* | 1450 | 581 | 393 | 82.97 | 55.86 | **-27.1** |
| Array | *NUDT15* | 1450 | 0 | 0 | 97.24 | 97.24 | 0.00 |
| Array | *SLCO1B1* | 1450 | 87 | 84 | 98.07 | 92.28 | **-5.79** |
| Array | *TPMT* | 1450 | 84 | 82 | 98.76 | 93.10 | **-5.66** |
| Array | *UGT1A1* | 1450 | 417 | 15 | 44.21 | 43.17 | **-1.03** |
| Array | *UGT2B7* | 1450 | 0 | 0 | 98.62 | 98.62 | 0.00 |
| Array | *VKORC1* | 1450 | 151 | 149 | 98.76 | 88.48 | **-10.28** |
| Integrated | *CFTR* | 1450 | 0 | 0 | 97.86 | 97.86 | 0.00 |
| Integrated | *CYP1A1* | 1450 | 101 | 99 | 99.52 | 92.69 | **-6.83** |
| Integrated | *CYP1A2* | 1450 | 0 | 0 | 100.00 | 100.00 | 0.00 |
| Integrated | *CYP2A13* | 1450 | 0 | 0 | 99.66 | 99.66 | 0.00 |
| Integrated | *CYP2A6* | 1450 | 0 | 0 | 85.86 | 85.86 | 0.00 |
| Integrated | *CYP2B6* | 1450 | 0 | 0 | 47.45 | 47.45 | 0.00 |
| Integrated | *CYP2C8* | 1450 | 0 | 0 | 99.72 | 99.72 | 0.00 |
| Integrated | *CYP2C9* | 1450 | 0 | 0 | 99.38 | 99.38 | 0.00 |
| Integrated | *CYP2C19* | 1450 | 5 | 5 | 97.79 | 97.45 | **-0.34** |
| Integrated | *CYP2D6* | 1446 | 2 | 0 | 51.80 | 51.80 | 0.00 |
| Integrated | *CYP2E1* | 1450 | 304 | 291 | 96.55 | 76.48 | **-20.07** |
| Integrated | *CYP2F1* | 1450 | 25 | 0 | 40.07 | 40.07 | 0.00 |
| Integrated | *CYP2J2* | 1450 | 0 | 0 | 99.17 | 99.17 | 0.00 |
| Integrated | *CYP2R1* | 1450 | 0 | 0 | 100.00 | 100.00 | 0.00 |
| Integrated | *CYP2S1* | 1450 | 0 | 0 | 99.93 | 99.93 | 0.00 |
| Integrated | *CYP2W1* | 1450 | 0 | 0 | 99.86 | 99.86 | 0.00 |
| Integrated | *CYP3A4* | 1450 | 13 | 13 | 99.93 | 99.03 | **-0.9** |
| Integrated | *CYP3A5* | 1450 | 0 | 0 | 96.83 | 96.83 | 0.00 |
| Integrated | *CYP3A7* | 1450 | 0 | 0 | 100.00 | 100.00 | 0.00 |
| Integrated | *CYP3A43* | 1450 | 101 | 101 | 99.59 | 92.62 | **-6.97** |
| Integrated | *CYP4F2* | 1450 | 268 | 265 | 99.24 | 80.97 | **-18.28** |
| Integrated | *DPYD* | 1450 | 107 | 104 | 98.97 | 91.79 | **-7.17** |
| Integrated | *GSTM1* | 740 | 0 | 0 | 18.24 | 18.24 | 0.00 |
| Integrated | *GSTP1* | 1450 | 0 | 0 | 98.07 | 98.07 | 0.00 |
| Integrated | *IFNL3* | 1450 | 0 | 0 | 99.86 | 99.86 | 0.00 |
| Integrated | *NAT1* | 1450 | 61 | 60 | 93.38 | 89.24 | **-4.14** |
| Integrated | *NAT2* | 1450 | 579 | 391 | 84.14 | 57.17 | **-26.97** |
| Integrated | *NUDT15* | 1450 | 0 | 0 | 98.28 | 98.28 | 0.00 |
| Integrated | *SLCO1B1* | 1450 | 91 | 89 | 97.17 | 91.03 | **-6.14** |
| Integrated | *TPMT* | 1450 | 79 | 78 | 99.45 | 94.07 | **-5.38** |
| Integrated | *UGT1A1* | 1450 | 417 | 15 | 44.21 | 43.17 | **-1.03** |
| Integrated | *UGT2B7* | 1450 | 0 | 0 | 98.83 | 98.83 | 0.00 |
| Integrated | *VKORC1* | 1450 | 151 | 148 | 99.03 | 88.83 | **-10.21** |

**Supplementary Table S6. Availability of CYP2F1 allele-defining variants across non-WGS datasets.** The table includes *CYP2F1* *2.001-defining variants, together with selected *3-, *5-, and *5.002-defining variants. Variant availability was assessed in normalized cohort-level VCFs. "Present" indicates an exact match to the expected normalized hg38 variant representation; "Not identified" indicates that no matching representation was found. WGS variant-level evidence was not assessed because WGS was analyzed from CRAM input.

| **Allele(s)** | **Defining variant** | **WES** | **Imputed array** | **Integrated call set** |
| --- | --- | --- | --- | --- |
| *2 | rs35107873 (G>A) | Not identified | **Present** | **Present** |
| *2 | rs34903717 (delC) | Not identified | **Present** | **Present** |
| *2 | rs2287940 (G>A) | Not identified | **Present** | **Present** |
| *2 | rs3833221 (insC) | **Present** | **Present** | **Present** |
| *2 | rs305968 (G>A) | **Present** | **Present** | **Present** |
| *2 | rs305974 (G>A) | Not identified | **Present** | **Present** |
| *2, *3 | rs75405062 (G>C) | Not identified | Not identified | Not identified |
| *2 | rs305975 (T>G) | **Present** | **Present** | **Present** |
| *2, *3 | rs305978 (C>A) | **Present** | Not identified | **Present** |
| *5 | rs144315434 (T>C) | Not identified | Not identified | Not identified |
| *5.002 | rs960127696 (C>T) | Not identified | Not identified | Not identified |
| *5.002 | rs991361920 (T>C) | Not identified | Not identified | Not identified |

**Supplementary Table S7. Synthetic VCF validation of nine *CYP2F1* *2.001-defining variants using Aldy v4.6.** The nine *CYP2F1* *2.001-defining variants comprised seven SNVs—rs35107873, rs2287940, rs305968, rs305974, rs75405062, rs305975, and rs305978—and two indels, rs34903717 and rs3833221. All REF alleles were validated against the reference FASTA. Aldy did not recover *2 from any tested alternate-allele configuration, including VCFs containing all nine variants in either unphased or phased form. The SNV-only and complete nine-variant VCFs were reported as *1/*1+rs305974+rs75405062, indicating a *1/*1 assignment with rs305974 and rs75405062 reported as additional variants rather than incorporated into a recognized *2 call. The indels-only VCF produced the same *1/*1 result as the negative control, indicating that rs34903717 and rs3833221 were not incorporated into the *CYP2F1* *2 assignment under the tested Aldy v4.6 VCF-mode conditions.

| **Synthetic VCF** | **Variants encoded as alternate** | **GT** | **Aldy result** |
| --- | --- | --- | --- |
| Negative control  (all reference) | None | 0/0 | *1/*1 |
| SNVs only | Seven *2.001-defining SNVs | 0/1 | *1/*1+rs305974+rs75405062 |
| Indels only | rs34903717 and rs3833221 | 0/1 | *1/*1 |
| SNVs + indels | Seven SNVs and two indels | 0/1 | *1/*1+rs305974+rs75405062 |
| SNVs + indels, phased | Seven SNVs and two indels | 0\|1 | *1/*1+rs305974+rs75405062 |

**Supplementary Table S8. Observed Aldy-reported *CYP2D6* structural motifs and their ancestry distribution in the WGS analytical reference.** Motifs were identified across all retained WGS solutions among the 1,446 participants with a callable *CYP2D6* WGS analytical-reference diplotype; a motif was included if it appeared in any retained solution. *36- and *68-containing motifs were classified as hybrid/conversion-associated and took precedence over the generic tandem and repeated-copy definitions. Among remaining motifs, different star-allele families joined by “+” were classified as mixed tandems, repeated components from the same parent star-allele family as duplication when two components were present or multiplication when three or more components were present, and *5 as deletion notation. A participant could contribute to more than one motif row, so motif-level counts are not additive and may exceed participant-level counts in Supplementary Table S9. These categories describe structural notation reported by Aldy and do not establish independently validated *CYP2D6* copy-number or structural accuracy in the absence of an orthogonal structural assay.

| **Structural motif group** | **Observed Aldy motif** | **Participants, n (%)** | **AFR/EAS/EUR/SAS, n** |
| --- | --- | --- | --- |
| *36-associated CYP2D6::CYP2D7 structure  (exon 9 conversion-containing; standalone or in a multi-copy arrangement) | *36.ALDY+*10 | 75 (5.19) | 0/74/0/1 |
|  | *1+*36.ALDY | 2 (0.14) | 0/2/0/0 |
|  | *10+*36.ALDY+*10 | 2 (0.14) | 0/2/0/0 |
|  | *36.ALDY | 8 (0.55) | 0/8/0/0 |
| CYP2D6::CYP2D7 hybrid  (*68; standalone or in tandem) | *68+*4.021 | 33 (2.28) | 0/0/33/0 |
|  | *68+*4 | 16 (1.11) | 0/0/15/1 |
|  | *68:2+*4.021 | 15 (1.04) | 0/0/14/1 |
|  | *68:2+*4 | 4 (0.28) | 0/0/4/0 |
|  | *1+*68:2 | 2 (0.14) | 1/0/1/0 |
|  | *68 | 7 (0.48) | 0/0/7/0 |
|  | *68:2 | 2 (0.14) | 0/0/2/0 |
| CYP2D6::CYP2D7  hybrid duplication (*68) | *68+*68 | 1 (0.07) | 0/0/0/1 |
| Mixed tandem | *1+*2 | 2 (0.14) | 0/0/1/1 |
|  | *79+*2 | 2 (0.14) | 0/0/2/0 |
|  | *1+*122 | 1 (0.07) | 0/1/0/0 |
|  | *1+*4.021 | 1 (0.07) | 0/0/1/0 |
|  | *1+*41 | 1 (0.07) | 0/0/1/0 |
|  | *1+*77 | 1 (0.07) | 0/0/1/0 |
|  | *10+*83.ALDY | 1 (0.07) | 0/1/0/0 |
|  | *2+*10 | 1 (0.07) | 0/1/0/0 |
|  | *39+*119 | 1 (0.07) | 1/0/0/0 |
|  | *41+*76 | 1 (0.07) | 0/0/1/0 |
|  | *76+*1 | 1 (0.07) | 0/0/1/0 |
| Multiplication | *4C+*4C+*4C+*4C | 2 (0.14) | 2/0/0/0 |
|  | *1+*1+*1 | 1 (0.07) | 0/0/1/0 |
|  | *10+*10+*10 | 1 (0.07) | 0/0/1/0 |
|  | *4C+*4C+*4C | 1 (0.07) | 1/0/0/0 |
| Duplication | *1+*1 | 23 (1.59) | 2/1/19/1 |
|  | *2+*2 | 17 (1.18) | 7/1/8/1 |
|  | *4C+*4C | 10 (0.69) | 9/0/1/0 |
|  | *10+*10 | 7 (0.48) | 0/6/1/0 |
|  | *4+*4N.ALDY | 6 (0.41) | 0/0/6/0 |
|  | *4+*4 | 3 (0.21) | 0/0/3/0 |
|  | *33+*33 | 2 (0.14) | 0/0/2/0 |
|  | *35+*35 | 2 (0.14) | 0/0/2/0 |
|  | *106+*106 | 1 (0.07) | 1/0/0/0 |
|  | *17+*17 | 1 (0.07) | 1/0/0/0 |
|  | *4+*4.021.ALDY | 1 (0.07) | 0/0/1/0 |
|  | *4+*4C | 1 (0.07) | 0/0/1/0 |
|  | *4C+*4M | 1 (0.07) | 0/0/1/0 |
| *5 deletion | *5 | 103 (7.12) | 12/18/63/10 |

**Supplementary Table S9. Participant-level *CYP2D6* structural categories and exact diplotype concordance across WES, imputed-array, and integrated call sets.** Percentages were calculated among the 1,446 participants with a callable *CYP2D6* WGS analytical-reference diplotype. Structural indicators were evaluated across all retained WGS solutions using an any-solution rule; a participant was assigned to the explicit-structural stratum if a structural indicator appeared in any retained solution. Of the 1,446 participants, 1,441 (99.7%) had one retained WGS solution and 5 (0.3%) had multiple retained solutions. Mutually exclusive categories were then assigned according to the following priority hierarchy: hybrid/conversion-associated allele, other mixed tandem configuration, same-allele multiplication, same-allele duplication, *5 deletion notation only, and no explicit structural indicator. The 159 hybrid/conversion-associated participants comprised 82 with *36-associated notation and 77 with *68-associated notation, with no overlap. Concordance columns report the primary any-match analysis; strict concordance for the same structural-notation strata is reported in Table 4. Absence of explicit structural notation does not indicate verified normal copy number, and these categories describe Aldy-reported notation rather than independently validated copy-number or structural status. The no-explicit-structural-indicator stratum is repeated in the mutually exclusive hierarchy for completeness.

| **Analysis level** | **Participant-level category** | **Participants,**  **n (%)** | **WES,**  **n/N (%)** | **Imputed array,**  **n/N (%)** | **Integrated, n/N (%)** |
| --- | --- | --- | --- | --- | --- |
| Overall analytical reference | Callable CYP2D6 WGS analytical reference | 1,446 (100.0) | 750/1,446 (51.9) | 790/1,446 (54.6) | 749/1,446 (51.8) |
| Structural-notation strata | Any explicit structural indicator | 344 (23.8) | 0/344 (0.0) | 0/344 (0.0) | 0/344 (0.0) |
|  | No explicit structural indicator | 1,102 (76.2) | 750/1,102 (68.1) | 790/1,102 (71.7) | 749/1,102 (68.0) |
| Mutually exclusive hierarchy | Hybrid- or conversion-associated allele present | 159 (11.0) | 0/159 (0.0) | 0/159 (0.0) | 0/159 (0.0) |
|  | —of which: *36-associated | 82 (5.7) | 0/82 (0.0) | 0/82 (0.0) | 0/82 (0.0) |
|  | —of which: *68-associated | 77 (5.3) | 0/77 (0.0) | 0/77 (0.0) | 0/77 (0.0) |
|  | Other mixed tandem configuration | 8 (0.6) | 0/8 (0.0) | 0/8 (0.0) | 0/8 (0.0) |
|  | Same-allele multiplication | 5 (0.3) | 0/5 (0.0) | 0/5 (0.0) | 0/5 (0.0) |
|  | Same-allele duplication | 69 (4.8) | 0/69 (0.0) | 0/69 (0.0) | 0/69 (0.0) |
|  | *5 deletion notation only | 103 (7.1) | 0/103 (0.0) | 0/103 (0.0) | 0/103 (0.0) |
|  | No explicit structural indicator | 1,102 (76.2) | 750/1,102 (68.1) | 790/1,102 (71.7) | 749/1,102 (68.0) |

**Supplementary Figures**

**Supplementary Figure S1. Extended union-of-FAIL analysis and ancestry-stratified star-allele frequencies across all pharmacogenes.** For each gene, union-of-FAIL samples were individuals with a callable WGS analytical reference for whom at least one non-WGS platform—WES, imputed array, or the integrated call set—had no retained diplotype matching any retained WGS solution. Panel (A) shows Sankey diagrams of diplotype flows across WES, array, the integrated call set, and WGS, with link widths proportional to sample counts. Panel (B) shows ancestry-stratified WGS star-allele frequencies. Panel (C) summarizes platform-specific discordance and integration benefit, loss, and net change relative to WES and array. Low-frequency diplotypes were grouped as “Other,” and “NoCall” denotes a missing or unavailable non-WGS result. Sankey colors distinguish diplotype categories and do not indicate concordance status.
